# Construction of cyclic enones via gold-catalyzed oxygen transfer reactions

**DOI:** 10.3762/bjoc.7.71

**Published:** 2011-05-13

**Authors:** Leping Liu, Bo Xu, Gerald B Hammond

**Affiliations:** 1 Department of Chemistry, University of Louisville, 2320 South Brook Street, Louisville, KY 40292, USA

**Keywords:** alkyne–carbonyl metathesis, cyclic enones, gold-catalyzed, oxonium, oxygen transfer

## Abstract

During the last decade, gold-catalyzed reactions have become a tour de force in organic synthesis. Recently, the gold-, Brønsted acid- or Lewis acid-catalyzed oxygen transfer from carbonyl to carbon–carbon triple bond, the so-called alkyne–carbonyl metathesis, has attracted much attention because this atom economical transformation generates α,β-unsaturated carbonyl derivatives which are of great interest in synthetic organic chemistry. This mini-review focuses on the most recent achievements on gold-catalyzed oxygen transfer reactions of tethered alkynones, diynes or alkynyl epoxides to cyclic enones. The corresponding mechanisms for the transformations are also discussed.

## Review

**Scheme 1:**
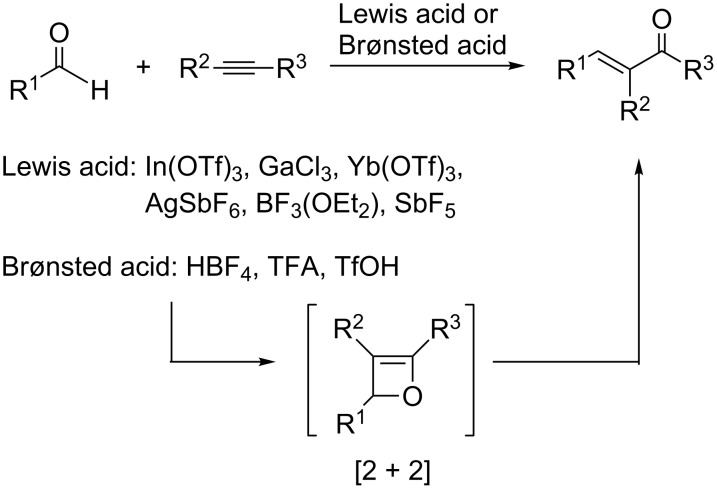
Lewis acid or Brønsted acid-catalyzed alkyne–carbonyl metathesis and a proposed [2 + 2] intermediate.

α,β-Unsaturated carbonyl derivatives are not only important building blocks in synthetic organic chemistry, but are also a significant motif in natural products and biologically active compounds [1–8]. The construction of the conjugated enone substructure has attracted the interest of synthetic chemists for decades. Among numerous methodologies, aldol condensations and Wittig-type reactions have been widely utilized [9–18]. Recently, it was found that conjugated enones could be generated from the oxygen transfer from a carbonyl group to a carbon–carbon triple bond, the so-called alkyne–carbonyl metathesis. This methodology has sparked the attention of the synthetic community, because it could serve as an efficient and atom-economic alternative to the Wittig reaction by the formation of a new carbon–carbon double bond and the simultaneous installation of a carbonyl group. In this regard, several Lewis or Brønsted acid-catalyzed intermolecular or intramolecular alkyne–carbonyl metatheses have been extensively studied (Scheme 1) [19–27].

During the early years of this century, organic chemists became aware that gold salts or complexes were highly active catalysts in homogeneous catalysis because of the strong π- and σ-electrophilicity of gold [28–33]. Since then, the number of new gold-catalyzed reactions reported in the literature has increased substantially and gold catalysis has become one of the hottest research fields in synthetic organic chemistry [34–42]. Due to their unique alkynophilicity, gold catalysts are especially suited to the activation of carbon–carbon triple bonds.

### Gold-catalyzed formation of cyclic enones from alkynyl ketones

**Scheme 2:**
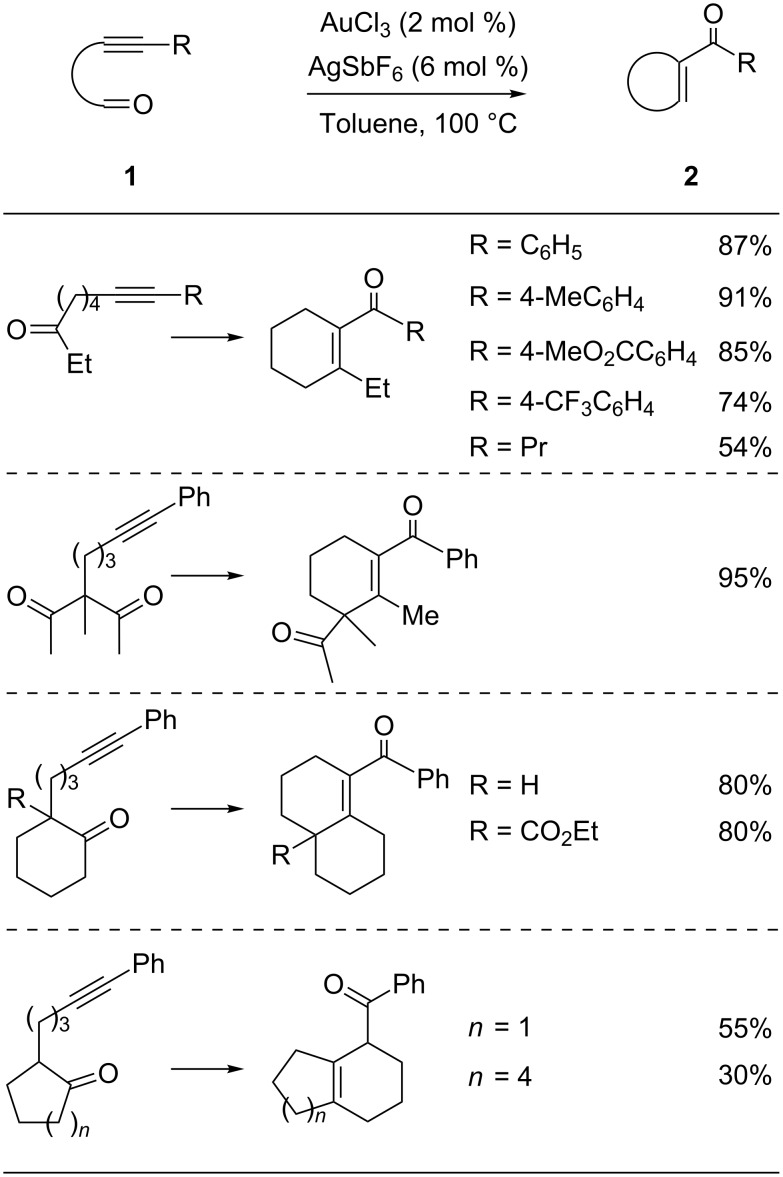
Gold-catalyzed cyclization of internal alkynyl ketones.

Yamamoto and co-workers were the first to report the gold-catalyzed formation of conjugated cyclic enones under mild conditions using tethered alkynyl ketones as substrates (Scheme 2) [43]. Both, aromatic and aliphatic groups substituted on alkynyl ketones **1** were investigated in this reaction, and the corresponding enone products **2** were isolated in good yields. They employed the alkyne–carbonyl metathesis in the preparation of fused ring systems and obtained two six-membered bicyclic products. However, if the original ring was five- or eight-membered, the reaction produced β,γ-unsaturated bicyclic enones rather than their α,β-unsaturated counterparts.

**Scheme 3:**
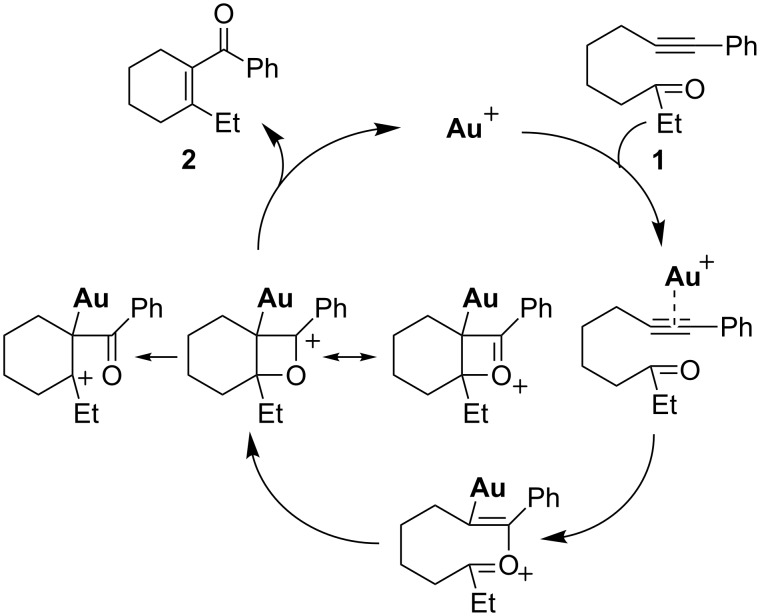
Proposed [2 + 2] mechanism for the cyclization of alkynyl ketones.

Yamamoto and co-workers proposed a [2 + 2] mechanism for their gold-catalyzed cyclization of alkynyl ketones (Scheme 3). In their mechanism, the carbonyl group attacks the gold activated triple bond to form an oxonium intermediate, which then generates an oxetenium intermediate. After several electron transfer steps, the cyclic enone product is formed. A similar [2 + 2] pathway has also been invoked for the Brønsted acid- or Lewis acid-mediated intramolecular and intermolecular alkyne–aldehyde metatheses.

**Scheme 4:**
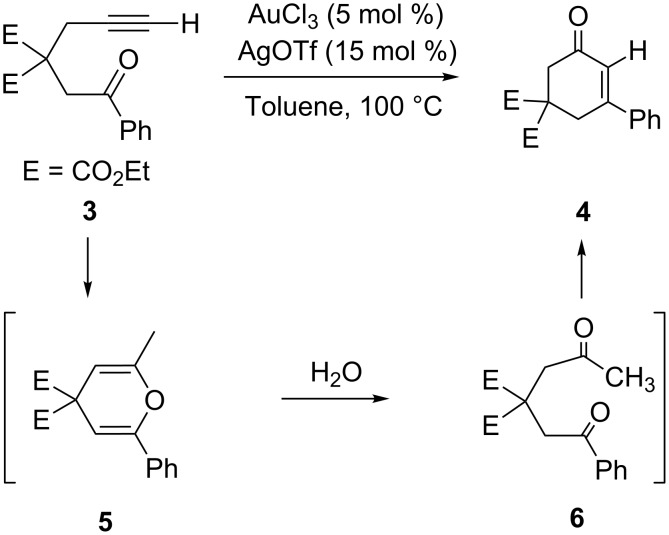
Gold-catalyzed cyclization of terminal alkynyl ketones.

If terminal alkynyl ketone **3** is employed as the substrate, the reaction still furnishes α,β-unsaturated cyclic enone **4**, but it necessitates a larger catalyst load (Scheme 4). By carefully monitoring of the reaction, it was found that intermediate **5** was formed together with a mixture of a hydrolyzed derivative of **6** and the final product **4**. The isolated intermediate **5** could be transformed into a mixture of **6** and **4** under the reaction conditions, finally yielding **4** via intramolecular aldol condensation.

**Scheme 5:**
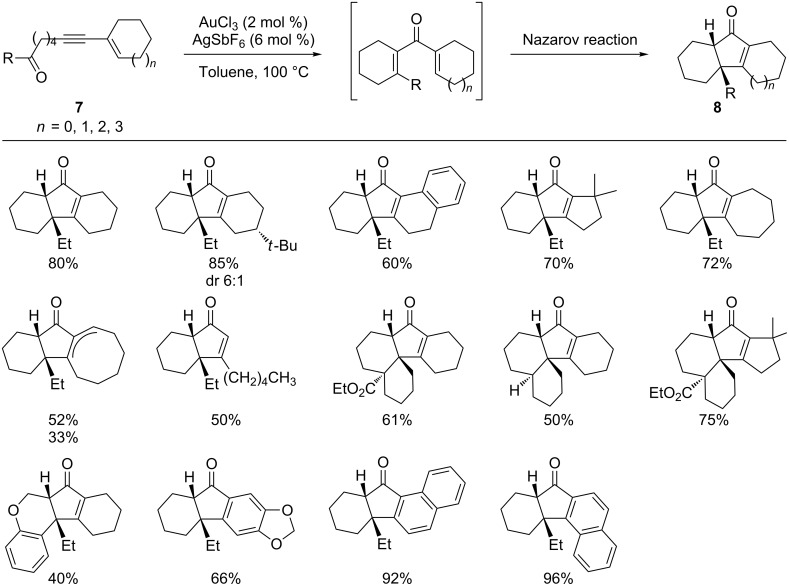
Gold-catalyzed tandem oxygen transfer/Nazarov cyclizations.

This gold-catalyzed cyclization of alkynyl ketones to enones was successfully utilized in a cascade reaction by the same authors (Scheme 5) [44]. Using enynones **7** as the substrate, the gold-catalyzed tandem alkyne–carbonyl metathesis/Nazarov reaction generated a number of intriguing fused bicyclic, tricyclic and tetracyclic derivatives of **8** in moderate to good yields and excellent diastereoselectivity. In this case, the gold catalyst exhibited a dual role, namely the activation of alkyne and carbonyl moieties.

**Scheme 6:**
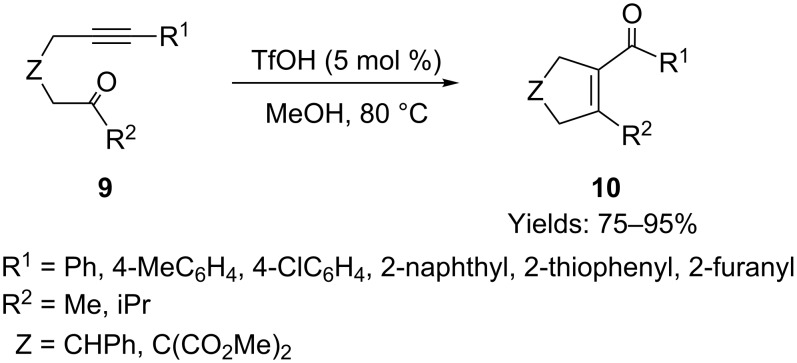
TfOH-mediated cyclization of alkynyl ketones.

Yamamoto and co-workers attempted to utilize their protocol to build five-membered cyclic enones, however, when they employed alkynyl ketone **9** as the substrate, the gold catalyst did not show good activity, and less than 30% of the desired product **10** was formed [45]. After optimizing the reaction conditions, the authors found that TfOH was the best catalyst for this oxygen transfer reaction in methanol (Scheme 6). This TfOH-mediated cyclization was applied to the synthesis of various fused tricyclic and tetracyclic derivatives of **10**.

**Scheme 7:**
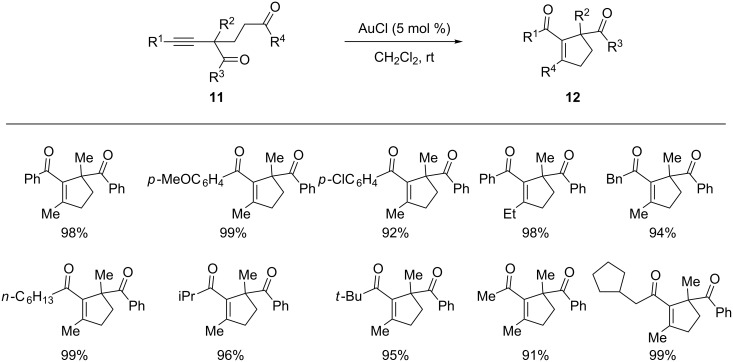
Gold-catalyzed cyclizations of 2-alkynyl-1,5-diketones.

Hammond and co-workers found that the gold-catalyzed oxygen transfer reaction proceeded very smoothly when using alkynyldiketone **11** as the substrate (Scheme 7) [46]. Indeed, this reaction was complete in 5 minutes at room temperature to give the five-membered cyclic enones **12** cleanly and in excellent yields. The large reactivity difference between substrates **9** and **11** prompted the authors to propose an alternative [4 + 2] mechanism for this transformation, rather than the previously proposed and well-accepted [2 + 2] pathway for oxygen transfer reactions.

**Scheme 8:**
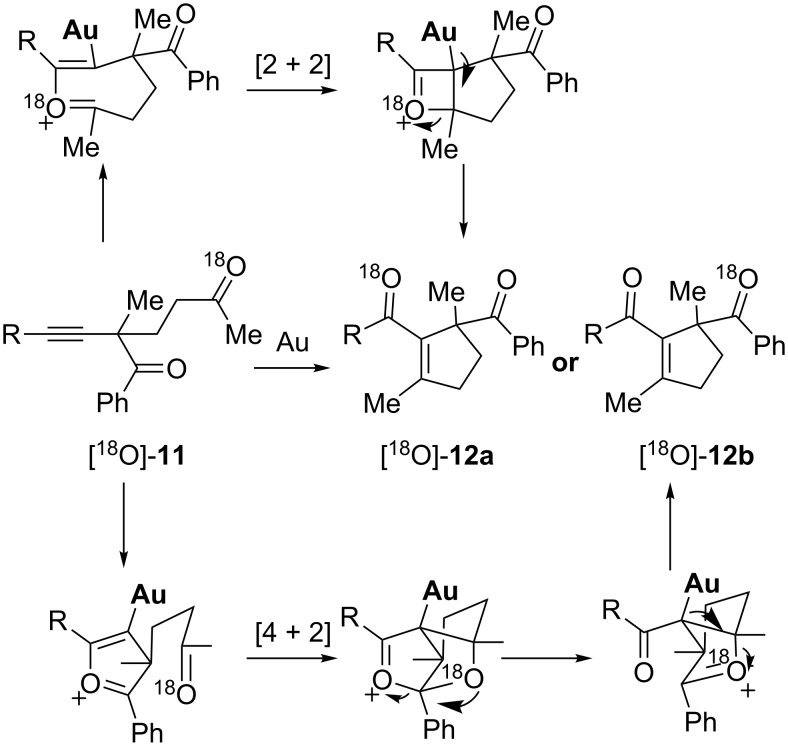
Designed isotopic labeling experiment for mechanistic studies.

An isotopic labeling experiment was designed to elucidate the pathway responsible for the gold-catalyzed intramolecular oxygen transfer of 2-alkynyl-1,5-diketones (Scheme 8). By introducing an ^18^O atom into one of the carbonyls of the substrate, and using the ^13^C NMR spectra of the substrate and product to locate the ^18^O atom, the authors hoped to elucidate the more favorable mechanistic pathway. The alkynyldiketone [^18^O]-**11** was chosen as a model substrate. If the reaction follows a [2 + 2] route then ^18^O would end up on the left carbonyl group in [^18^O]-**12a** (Scheme 8, top), whereas it would be incorporated on the benzoyl group in [^18^O]-**12b** if the reaction follows a [4 + 2] pathway (Scheme 8, bottom).

**Scheme 9:**
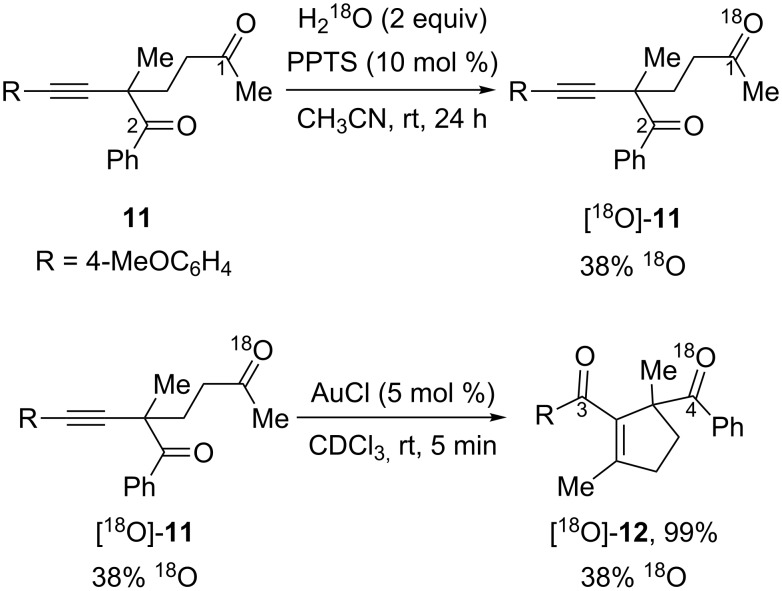
^18^O isotopic experiments.

The result of this isotopic experiment is outlined in Scheme 9. Substrate [^18^O]-**11** was synthesized from the ^18^O exchange of compound **11** with H_2_^18^O under acidic conditions, and its ^13^C NMR spectrum showed that the ^18^O exchange happened only at the methyl carbonyl group (carbon 1). This substrate was subjected to the gold-catalyzed oxygen transfer reaction conditions and the product [^18^O]-**12** was obtained in quantitative yield without any ^18^O loss. It was later found that the ^18^O was only incorporated into the benzoyl group (carbon 4) in product [^18^O]-**12**, as determined from its ^13^C NMR spectrum. The absence of any detectable ^18^O incorporation at carbon 3 demonstrates that the [2 + 2] pathway is disfavored, and instead it is the [4 + 2] pathway that is the favored mechanism in the gold-catalyzed intramolecular oxygen transfer of 2-alkynyl-1,5-diketones.

**Scheme 10:**
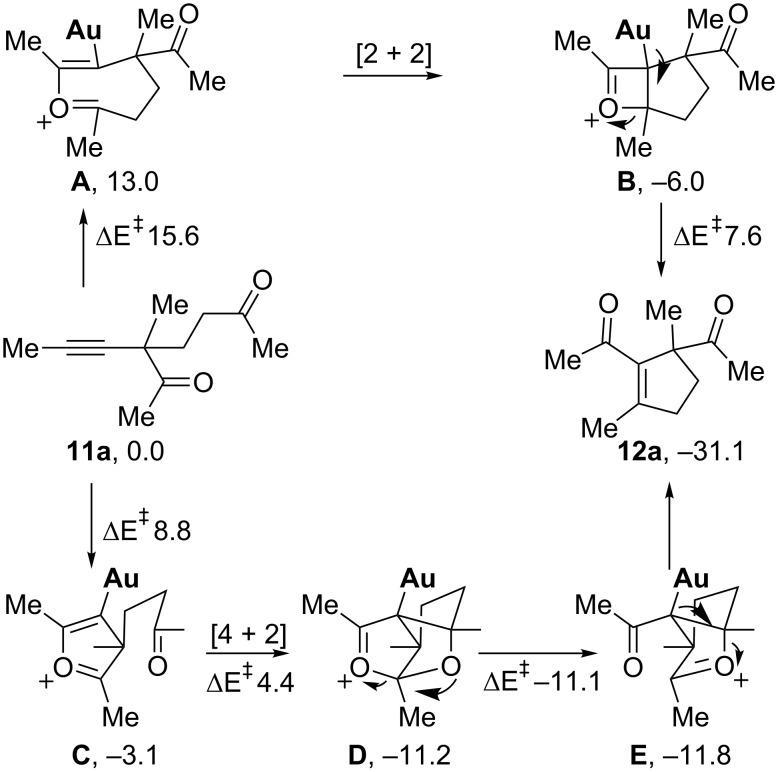
B2PLYP/6-311+G(d,p)//B2PLYP/6-31G(d) computed reaction profile, relative energies in kcal/mol.

The discovery of a [4 + 2] cycloaddition of a furanium intermediate to a carbonyl group was further verified by quantum chemical calculations. The competing [2 + 2] and [4 + 2] reaction coordinates were computed for the simplified substrate **11a**, shown in Scheme 10. In accordance with the experimental findings, the [4 + 2] pathway is found to be the more favorable. The rate-limiting step in each pathway is the intramolecular nucleophilic addition to the Au-coordinated alkyne – the barrier for this step is computed to be 6.8 kcal/mol lower for the formation of the five-membered ring oxonium intermediate **C** than for the seven-membered ring oxonium **A**. This energetic preference is also observed in the stabilities of the oxoniums themselves, with **C** considerably more stable by 16.1 kcal/mol. The subsequent transformations are all computed to be feasible, with the barrier to [4 + 2] cyclization lying only 4.4 kcal/mol above the starting complex. Further calculations on the barrier for transition states were also consistent with the rapid conversion that was observed in the experiments. Overall, the large energetic preference of the intermediates and transition states for the [4 + 2] pathway over the [2 + 2] pathway supports the postulate that the [4 + 2] pathway is dominant in the gold-catalyzed oxygen transfer of 2-alkynyl-1,5-diketones, which is exactly in accordance with the ^18^O isotopic experiments.

**Scheme 11:**
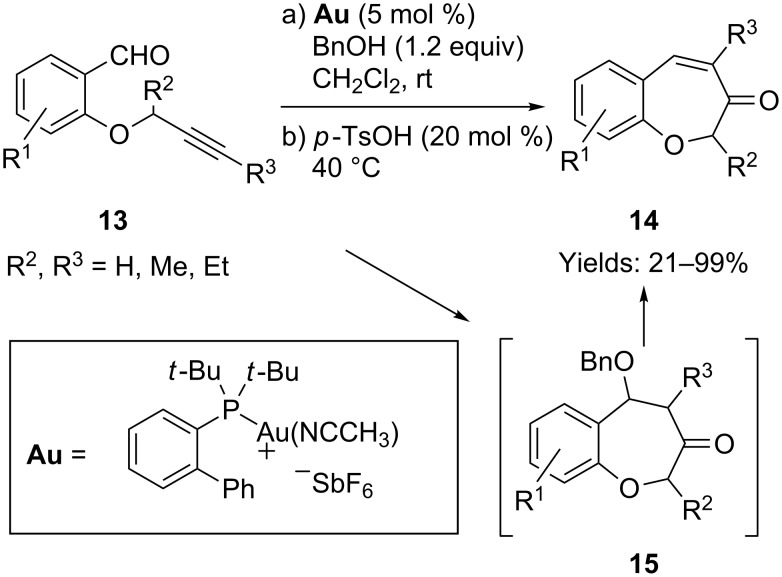
Gold-catalyzed cyclization of tethered alkynyl arylaldehydes.

Chan and co-workers developed a gold-catalyzed tandem intramolecular rearrangement of alkynyl arylaldehydes **13** to benzoxepinones **14** with good regioselectivity (Scheme 11) [47]. This transformation was effectively promoted by the addition of benzyl alcohol and the sequential addition of *p*-toluenesulfonic acid. However, in the absence of *p*-toluenesulfonic acid, benzyl ether **15** was isolated as the major product. The latter was considered to be an intermediate in the reaction and moreover, the isolated compound **15** could be transformed into the final product **14** under the mediation of *p*-toluenesulfonic acid.

### Gold-catalyzed formation of cyclic enones from diynes

**Scheme 12:**
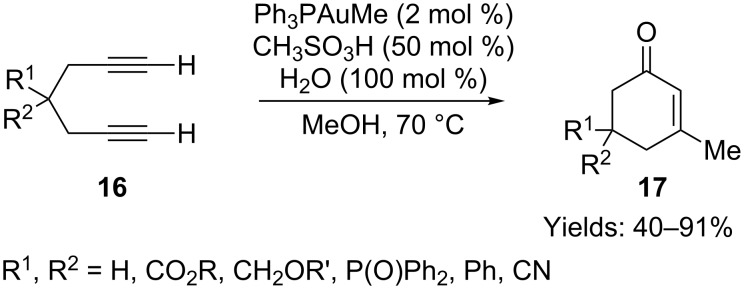
Gold-catalyzed cyclization of terminal diynes.

Zhang and co-workers reported gold-catalyzed cyclizations to cyclohexenones **17**, employing terminal 1,6-diynes **16** as substrates in the presence of a Brønsted acid and 1 equiv of water (Scheme 12) [48]. None of the desired products were obtained in the absence of the gold catalyst, the Brønsted acid or water. Interestingly, when the diacid 1,6-diyne (R^1^ = R^2^ = COOH) was employed in the reaction, only the esterified product (R^1^ = R^2^ = COOMe) was isolated, albeit in low yield. The authors also carried out this gold-catalyzed transformation in an ionic liquid [49]. This modification enabled the separation of the gold catalyst from the organic mixture and the recovered gold catalyst in the ionic liquid was re-used as many as five times without loss of activity.

**Scheme 13:**
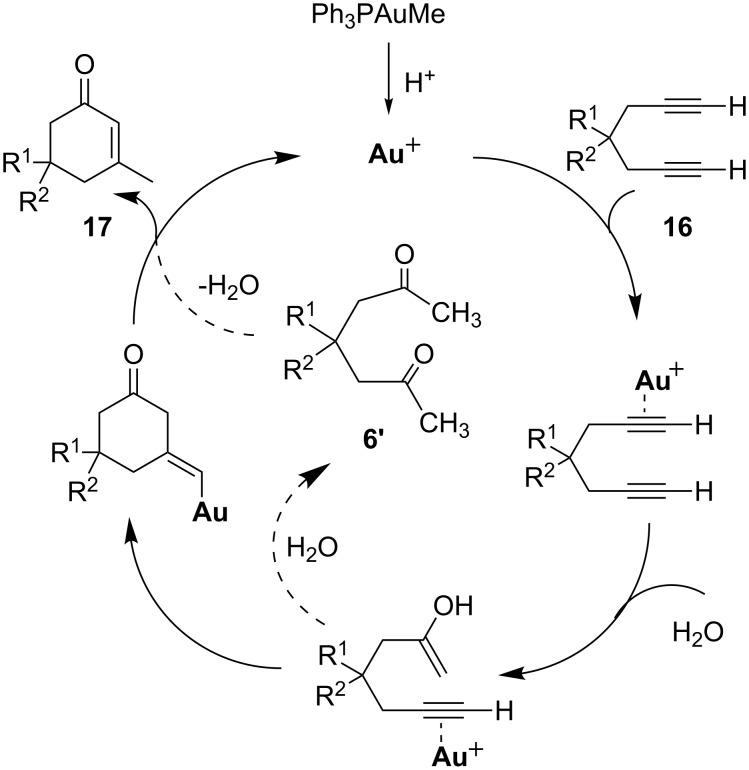
Proposed hydrolysis/cyclization mechanism.

A hydrolysis/cyclization mechanism was proposed for the transformation (Scheme 13). Although this mechanism is plausible, another option for the cyclization step might exist. One of the key intermediates in the catalytic cycle is the hydrolyzed product – the alkynyl ketone from hydrolysis of one triple bond – which is the same as the substrate that was employed by Yamamoto and co-workers. Thus, a similar diketone intermediate **6'** could also have been formed before being transformed into the final product via intramolecular aldol condensation.

**Scheme 14:**
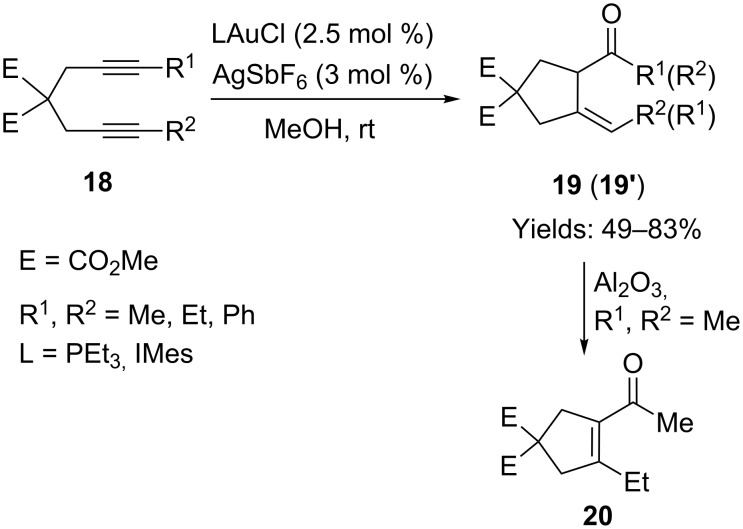
Gold-catalyzed cyclization of internal diynes.

Fiksdahl and co-workers investigated a similar gold-catalyzed transformation of internal 1,6-diynes **18** in methanol at room temperature (Scheme 14) [50–51]. Interestingly, a non-conjugated five-membered cyclic enone **19** was isolated as the product, instead of the conjugated cyclohexenone that was obtained from terminal 1,6-diynes. However, the scope of this transformation was limited to just a few substituent variations on the alkynes. When both R^1^ and R^2^ were ethyl groups, this cyclization was dramatically retarded and only traces of the desired product were obtained. Under the mediation of aluminium oxide, this non-conjugated cyclopentylidene ketone product isomerized to the conjugated cyclopentenyl ketone **20**.

**Scheme 15:**
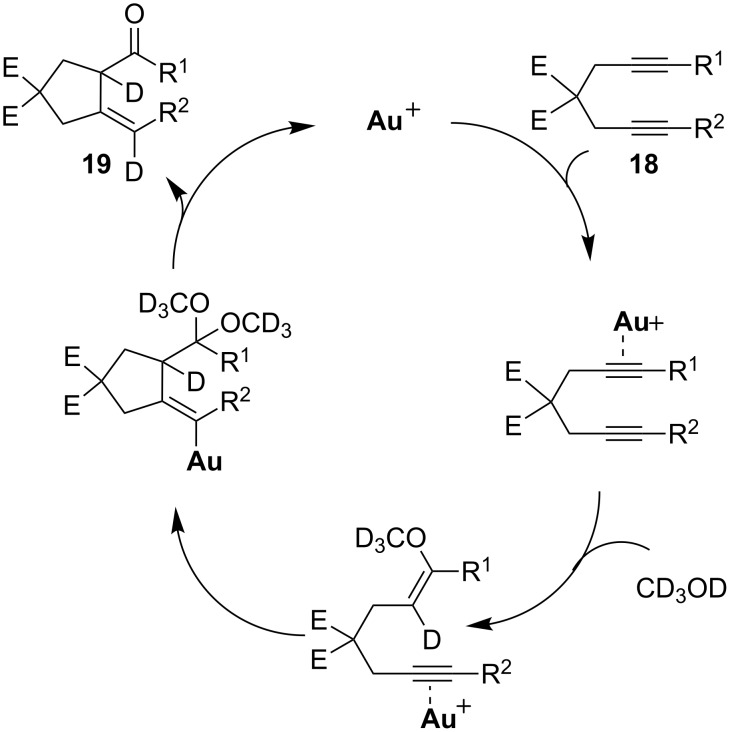
Proposed solvolysis/cyclization mechanism.

The authors proposed a solvolysis/cyclization mechanism for this gold-catalyzed cyclization, which was supported by a deuterium isotopic experiment (Scheme 15). Two molecules of methanol were involved in the transformation and a dimethoxyketal intermediate was formed: The final product was derived from the hydrolysis of this ketal intermediate. When *d*_4_-methanol was used as the solvent, a highly deuterated product was isolated, which provided strong support for the proposed mechanism.

### Gold-catalyzed formation of cyclic enones from alkynyl epoxides

**Scheme 16:**
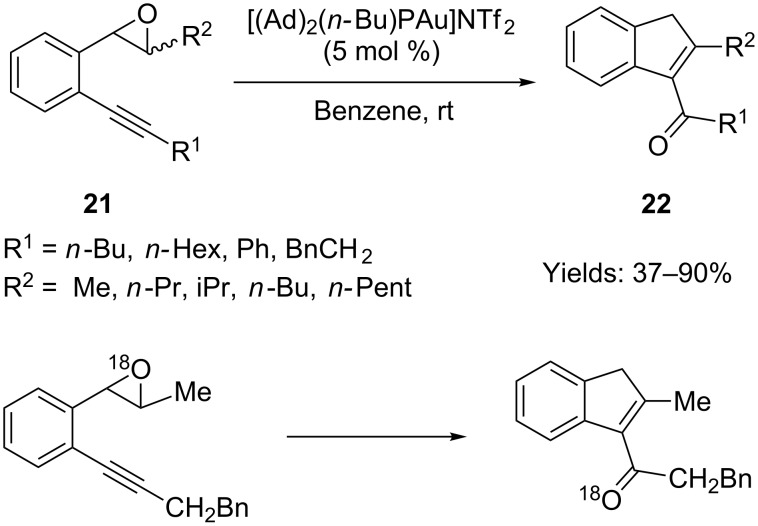
Gold-catalyzed cyclization of alkynyl epoxides and the ^18^O isotopic labeling experiment.

Hashmi and co-workers synthesized a number of 2-alkynyl aryl epoxides **21** intended to be used as substrates for a gold-catalyzed rearrangement to naphthols. Surprisingly, acylindene **22** turned out to be the product of this reaction, rather than the expected naphthol (Scheme 16) [52]. However, when a bulky group was substituted on the triple bond, this gold-catalyzed transformation was completely suppressed. Moreover, none of the desired product could be obtained when a terminal alkyne, a TMS-substituted alkyne, or even an ester-substituted epoxide was used as the starting material.

**Scheme 17:**
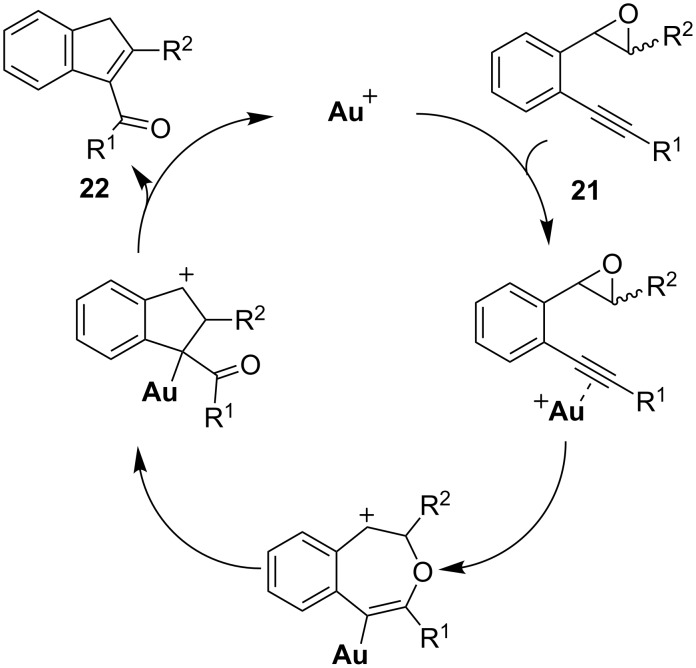
Proposed oxygen transfer mechanism.

An ^18^O isotopic experiment helped the authors to propose an intramolecular oxygen transfer mechanism for the above transformation (Scheme 17). When employing the ^18^O incorporated substrate in the reaction, the authors found that the isolated product still contained the isotopic atom which excludes the involvement of external water in the reaction. A cross-over experiment with a mixture of two substrates (one with ^18^O, the other without) was also conducted, and no ^18^O scramble was found in the products, which clearly supported the intramolecular nature of the oxygen transfer.

**Scheme 18:**
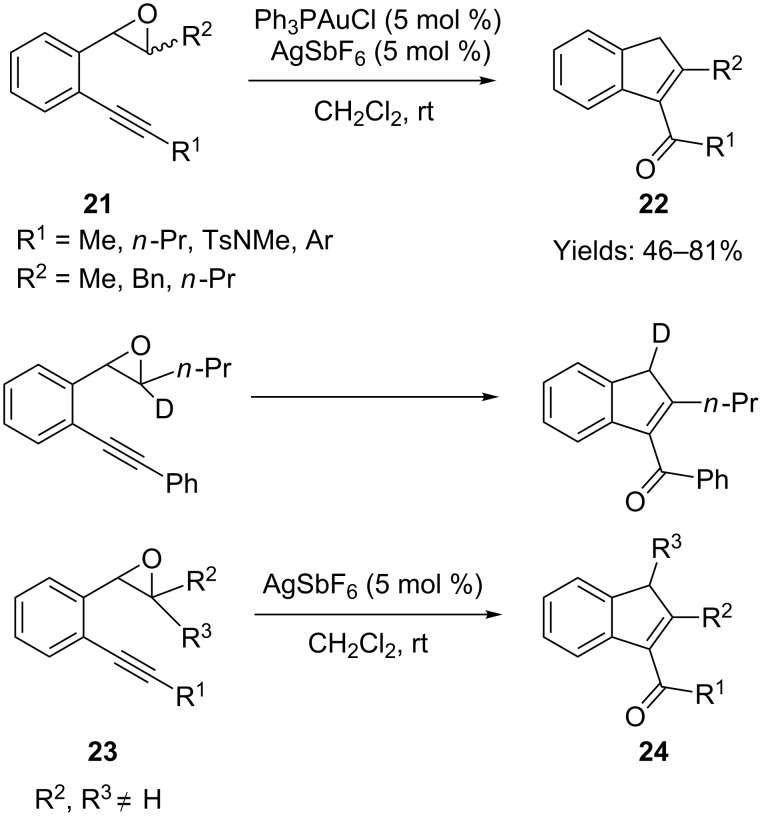
Gold or silver-catalyzed cyclization of alkynyl epoxides and the corresponding deuterium labeling experiment.

Liu and co-workers independently reported a very similar gold-catalyzed cyclization of 2-alkynyl aryl epoxide **21** to acylindene **22** (Scheme 18) [53]. A deuterium isotopic experiment was conducted to support the intramolecular oxygen transfer mechanism. However, when a trisubstituted epoxide **23** was employed in the reaction, the gold catalyst did not promote the transformation. By contrast, when AgSbF_6_ was used as the catalyst, the 1,2-alkyl shifted product **24** was obtained.

## Conclusion

This short review compiles recently reported gold-catalyzed oxygen transfer reactions used to build cyclic enones from tethered alkynyl ketones, 1,6-diynes or 2-alkynyl aryl epoxides. Most of these reactions take place under mild conditions and the corresponding products were isolated in good yields. The mechanisms for these transformations were also comparatively discussed. Similar Brønsted acid or other metal mediated transformations and their applications to cascade cyclizations were additionally described. Given gold’s strong π-electrophilicity, it is expected that novel applications of gold catalysts in reactions of alkynes, allenes, and even alkenes, will continue to attract the attention of synthetic chemists.
